# Dental Legislation and Its Necessities

**Published:** 1876-12

**Authors:** E. S. Holmes


### DENTAL REGISTER.

Vol. XXX.] DECEMBER, 1876. [No. 12.

### DENTAL LEGISLATION AND ITS NECESSITIES.

BY E. S. HOLMES, D. D. S.

*Read before the Michigan State Dental Society.*

In all communities of animated beings, there seems to be a demand for laws to govern and control actions, so as to prevent individual interests from compromising the general good and at the same time to protect personal rights from being crushed out by an assumption of public necessity. Germination, growth and existence are controlled by law, and the manifestations of the mind through the physical being, or social intercourse, must be subservient to wise and judicious laws in order to secure the greatest good of every member of society, and at the same time promote the weal of the whole body.

The authority to make and execute human law must necessarily be derived, either directly or remotely, from the governed. This power is sometimes permitted to rest in an individual, who presumes to possess a divine right to rule. But when the people decree to change their ruler, that divinity vanishes. At the present day the legislative and executive functions rest in different bodies. In our own country, legislators are chosen by the people, for the expressed purpose of establishing laws. This arrangement, it would seem, ought to insure popular laws, and if the people are intelligent, should also secure the enactment of judicious, equitable laws. In fact, the laws of a state are a good index of the general intelligence of the inhabitants of that state. In such a municipality the material interests of the community will be carefully guarded by legal enactments; not only in a general way, but by specific legislation will the individual interests of the people be carefully protected from the selfish designs of the crafty and unprincipled part of society.

These self-evident statements may be illustrated by a brief reference to a few of the wise and equitable laws of our own state.

In matters of trade and commerce, rigid laws, enforced by severe penalties, protect us from injury by the use of tainted meats. The druggist is surrounded by the strictest regulations in the dispensing of his poisons. The dealer in spirituous liquors has a check put upon his avarice by statutory provisions, and the pursuit and capture of game of fur, fin and feather, for the purpose of supplying one's self or family with healthful and toothsome food, is controlled by legal enactments, so as to prevent the extermination of animals "feræ naturæ," and preserve this important source of food supply and invigorating recreation.

In educational affairs we find the guardian arm of the law concerning the interests of our children, by keeping charlatanry and incompetency out of our public schools. No one is allowed to teach our children their a b c's even, without a certificate of competence from the constituted authorities, a diploma after having undergone a careful examination. The interests of the people of the state, in this respect, are carefully and wisely protected, as it would be notoriously improper to pay a teacher who was not thoroughly competent to perform the services for which he was employed.

Again, any one having business in the courts of this state, no matter how important his suit may be, is prohibited from employing a lawyer to manage the case, unless the lawyer shall have acquired a thorough professional education, and received his diploma, either from a chartered law school, or from the court in which he is to practice, as evidence of his ability to serve his client faithfully and successfully. Now these municipal regulations, doing all that statutory enactments can do to protect the people in person and property from hurt by the ignorant or unprincipled, are clearly wise and equitable provisions. They commend themselves to every wise and discreet citizen. They are approved by every philanthropist, by every one desiring the best interests of every inhabitant of the state, and the weal of the whole community.

This is not, and is not intended to be, a complete list of the legal enactments now in force in our state for protecting the interests of the people of the commonwealth, but is only given as an illustration of what has been done, and done wisely, for the purpose indicated. Now I wish to inquire: Would it not be an equal exhibition of wisdom on the part of our legislators, to extend the strong, protecting arm of the law over the people in regard to the practice of medicine and surgery, in all their departments, including of course, dental medicine and surgery—or dentistry? Perhaps it would be proper for me to state here, that in this paper I have no reference to what is usually called "mechanical dentistry," or the manufacture of artificial dentures, but I speak of dentistry as a conservative, curative art—as the own sister of medicine and surgery, in fact it is medicine and surgery applied to the cure of the diseases of the teeth and mouth, primarily; and, secondarily, to the cure of such diseases as are caused directly by the abnormal condition of the organs of mastication. Therefore, any comparison or argument in favor of the protection of the people from the evils of charlatanry and incompetence in the practice of general medicine or surgery will apply with equal force with reference to a law for the regulation of the practice of dentistry, and *vice versa.* It seems only necessary, then, to suggest the idea to secure the approval of every sound mind in the state. When we take into account the fact that the physician, the surgeon and the dentist have more to do with the health, comfort and life of the people than any other class in the community, it is simply inexplicable that laws for the protection of the people from the sad effects of incompetence, charlatanry and malpractice in the healing professions, were not the first to claim the attention of our legislators. No one is allowed to give rudimentary instruction to our children in the public schools, without having first secured a certificate from the legal authorities vouching for his fitness and abilities to perform the duties of his profession. But if these same children are stricken down with disease, either of the general system, or of the teeth and mouth, the most unprincipled and incompetent quack may be called to minister to them in their sufferings, or more probably to increase their agonies, or mutilate them for life, and perhaps hasten them to their graves. And the law gives no warning voice, lights no beacon to guide them in their choice of doctors, and utters no anthemas against empiricism. Are there not as good, as cogent reasons for a law to regulate the practice of the professions whose business it is to conserve the health of the people, as for the one now on our statute books, and wisely there, to regulate the practice of law? Is it not as important that the people should be protected from the ill effects of ignorance and unskillfulness in matters pertaining to health, comfort and life, as those having reference to property, or to selfish desires?

It was once remarked by a popular lawyer, who had unsuccessfully tried his hand at both medicine and divinity, in answer to a question by a friend, that he had "learned that mankind cared more for their wills than for soul and body both," which statement one who attends our courts of law will often see verified. But is this natural perverseness any reason why the strong arm of the law should not be invoked to protect the people against charlatanry in dentistry, as well as in the other professions of law and teaching? The answer to these questions must necessarily be given in the negative.

There is no real reason why the state should not furnish "legal protection from the evils of ignorance, incompetence and unskillfulness, in any one profession and not in another." And if there are any reasons for protection by laws regulating the practice of any profession, there are tenfold more and better reasons why such laws should be applied to the dental specialty of the occult healing art.

There is already a *necessity* for a law to regulate the practice of dentistry in this state. Dentists themselves would be benefited by such a law, for it would force them to become better educated and more skillful, but the greatest benefit would accrue to their patients and patrons, on account of that increased knowledge and ability that would enable the dentist to save them from disease, pain and discomfort. Let the people wake up to this necessity, and let us, as leaders of the people in this respect, importune our legislature for this wise and beneficent law, till they shall be obliged to acknowledge that what the people demand they will have done.

